# Successful rechallenge with cisplatin following cisplatin induced ischemic cerebrovascular accident in a patient with small cell lung cancer

**DOI:** 10.1002/ccr3.6469

**Published:** 2022-10-20

**Authors:** Farah Rashid, Nabil E. Omar, Hassan Aaekl, Ussama Al Homsi, Alaaeldin Shablak

**Affiliations:** ^1^ Department of Internal Medicine Hamad General Hospital, Hamad Medical Corporation Doha Qatar; ^2^ Pharmacy Department National Center for Cancer Care and Research, Hamad Medical Corporation Doha Qatar; ^3^ Department of Medical Oncology National Center for Cancer Care and Research, Hamad Medical Corporation Doha Qatar

**Keywords:** cerebrovascular accident, cisplatin, cisplatin induced stroke, small cell lung cancer

## Abstract

Cisplatin is a widely used platinum‐based chemotherapy agent. Its common adverse effects are neuropathy, nephrotoxicity, electrolyte abnormality, and rarely causing thrombotic vascular toxicity. We present a patient known to have small‐cell lung cancer who developed ischemic cerebrovascular accident (CVA) after receiving chemotherapy regimen including cisplatin.

## Graphical abstract

Cisplatin is rarely known to cause vascular toxicity resulting in thrombosis. Clinicians should be cautious of this side effect and act vigilantly if any patient presents with new onset neurological deficit or chest pain while he is on chemotherapy regimen consisting of cisplatin.

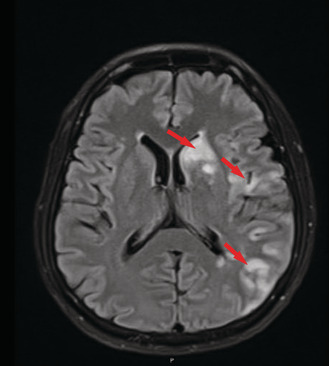

## 1 INTRODUCTION

Small cell lung cancer (SCLC) is the most aggressive type of lung cancer and represents 15% of all lung cancer.^1^ The median 5 years survival depends on the stage of the disease with an average of 7% for stage 4.^2^ Cigarette smoking remains to be the primary risk factor for developing SCLC.^3^ It is broadly divided into two categories: Limited Stage and Extensive stage. Generally, limited stage is treated with chemo‐radiotherapy while extensive stage with palliative chemotherapy only. Currently, Cisplatin and etoposide combination is the most widely used chemotherapy regimen to treat SCLC‐LS in combination with radiotherapy (RT).^4^ Cisplatin is a platinum‐based chemotherapy drug. It acts by crosslinking with the purine bases on DNA resulting in DNA damage and subsequently apoptosis of cancer cells. Its adverse effects include electrolyte imbalance, ototoxicity, and nephrotoxicity.^5^ Rarely, Cisplatin induces vascular toxicity with venous complications more common than arterial.^6^ Various mechanisms have been postulated to explain this effect including generation of a thrombotic state by inducing a high level of von Willebrand factor, decreasing the activity of protein C, and direct endothelial injury.^7^, ^8^, ^9^ We present a case of rare side effect of cisplatin in a patient with SCLC resulting in ischemic CVA due to arterial vascular thrombosis.

## 2 CASE PRESENTATION

**FIGURE 1:**
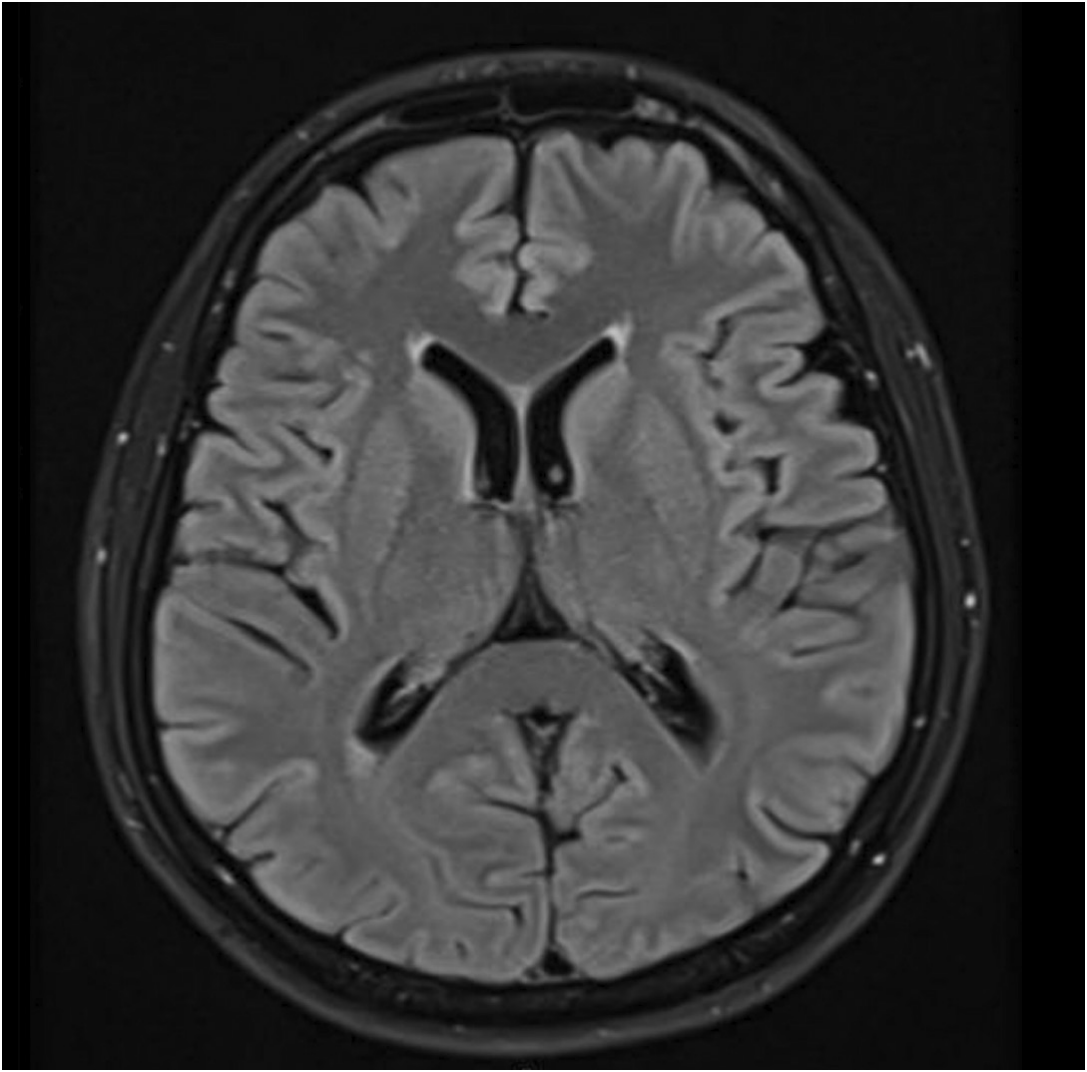
Initial MRI head showing normal study

A 45‐year‐old male patient with no previous medical illness presented to the emergency department with 2 months history of productive cough, associated with intermittent hemoptysis, weight loss, night sweats, pleuritic chest pain, and low‐grade fever. He had a smoking history for more than 10 years. He has no significant family history of stroke/cardiovascular disease, malignancy and prothrombic state. Chest X‐ray on admission showed right middle lobe consolidation. Afterward, CT scan of the chest documented right lower lobar consolidative opacity. Patient underwent bronchoscopy and right lower lobe mass was seen. Biopsy was taken and hence, diagnosed as SCLC. Staging PET scan and MRI head (Figure 1) were performed and revealed no distant metastasis. Patient cancer was staged as limited‐stage SCLC. His performance status was ECOG 2. In the Multidisciplinary team discussion, chemo‐radiation therapy was agreed on as a treatment modality.

**FIGURE 2:**
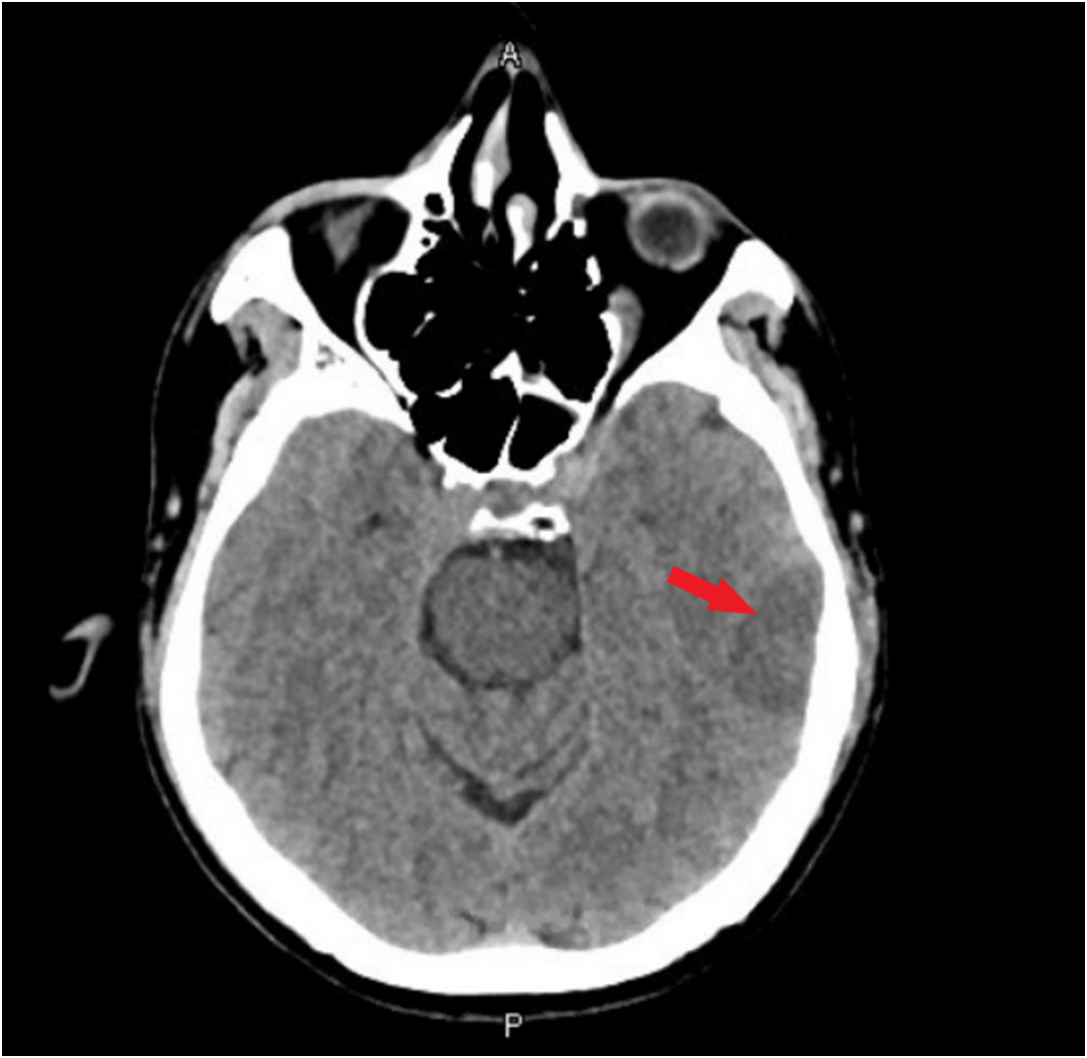
CT Head showing hypodense lesion in left temporal lobe (arrow) possible ischemic foci.

**FIGURE 3:**
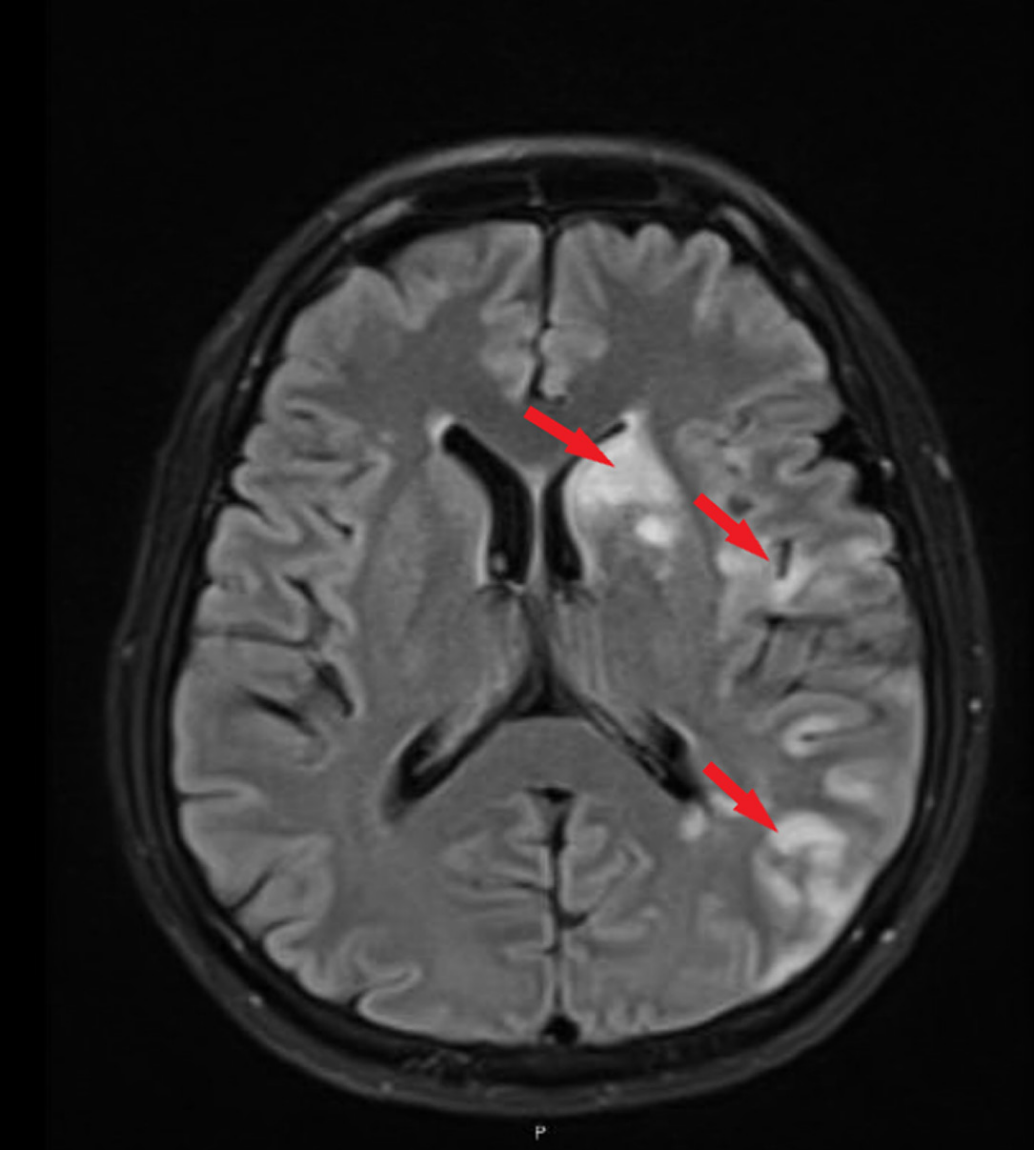
MRI Head showing multiple acute ischemic infarctions (arrows) in left basal ganglia and left cerebral hemisphere.

Chemotherapy regimen consisting of cisplatin 25 mg/m^2^ and Etoposide 100 mg/m^2^ were given intravenously on day one followed by Etoposide 100 mg/m^2^ intravenously for days 2 and 3. Radiotherapy was planned to be given with the second cycle. Four days after completing the first cycle, the patient presented with confusion, complete aphasia, and right‐sided weakness. Upon initial assessment, his vital signs were within normal limits, and he was alert and responsive. Neurological examination exhibited complete aphasia, no facial asymmetry, and motor power on the right side, upper limb 4/5, and lower limb 1/5 while on left side power was 5/5 in both upper and lower limbs. Reflexes were intact, the Babinski sign was negative, and hypertonicity was noted on the right side. CT scan of the head revealed possible ischemic foci on the left temporal lobe (Figure 2). Neurology was consulted and their impression was an embolic stroke and less likely to be metastatic disease. To confirm, MRI head was done and confirmed an acute infarct in the left cerebral hemisphere and basal ganglia predominantly left MCA territory infarct (Figure 3).

Multiple investigations were carried out to inspect for the cause of CVA. ECG showed normal sinus rhythm. Echocardiogram reported as normal. Holter monitor for 48 hours did not reveal an arrhythmia. Laboratory findings were within normal limit including lipid profile, Hba1c, and electrolyte panel including magnesium level. Patient was commenced on aspirin and statin. After a period of 2 weeks, patient's neurological condition improved with active physical and occupational therapy.

The oncological management was re‐discussed. After assessing the risk of developing another ischemic event with rechallenging versus the risk of developing the metastatic disease with suboptimal chemotherapy, it was finally decided based on the best interest of patient with curative intent to rechallenge him with cisplatin and etoposide. We proceeded with the second cycle of chemotherapy under cover of antiplatelet with close monitoring of the patient's neurovitals, the second cycle was tolerated well without developing any neurological deficit.

## 3 DISCUSSION

Cancer patients are prone to thromboembolic events because of the hypercoagulable state driven by malignancy.^10^ Furthermore, chemotherapy itself is a risk factor for thromboembolism (TE), which is the second leading cause of death in cancer patients.^11^ Many chemotherapy agents have been implicated in the development of thromboembolism. Cisplatin is a platinum‐based chemotherapy agent, which acts by DNA crosslinking and inducing apoptosis in cancer cells_._ Its side effect profile includes gastrointestinal upset, electrolyte abnormality, neurotoxicity, and nephrotoxicity.^5^ Interestingly, cisplatin is proposed to cause a higher incidence of vascular toxicity compared with other chemotherapy agents. However, the cisplatin‐induced vascular toxicities are likely to be venous rather than arterial.^6^

Previously, a retrospective study conducted on 10,963 cancer patients reported the incidence of ischemic CVA to be 0.137% in patients on chemotherapy. It was also noted that Platinum‐based compounds specifically cisplatin be the most used chemotherapy agent and middle cerebral artery was the most common artery to be involved. Moreover, 75% of these CVA occur within the first 10 days of the chemotherapy session and 60% occurred after the first cycle.^12^ Several mechanisms have been postulated to explain vascular toxicity. These include cisplatin developing a hypercoagulable state by increasing the level of von Willebrand factor^7^ and decreasing protein C activity.^8^ Additionally, it has been studied that cisplatin infusion releases free radicals which kindle direct endothelial injury, initiating a coagulation cascade leading to platelet aggregation and eventually, thrombosis.^9^ Also, the hypomagnesemia triggered by cisplatin further increase vascular smooth muscle contraction contributing to vascular thrombosis.^13^

In our case, extensive investigations were carried out to look for a probable cause of ischemic CVA in our patient. Apart from being a chronic smoker, and having a malignancy make him prone to thrombotic state in the body but given the fact that he is young, with no previous medical illness, acute incidence of CVA that happened after starting the chemotherapy would be the most likely explanation, making cisplatin to be the potential contributing factor giving its well‐established vascular toxicities. On the contrary, there is no evidence in literature that suggest etoposide can cause arterial toxicity.

So far, only one case has been published of rechallenging a patient who developed CVA after initiating cisplatin‐based chemotherapy.^14^ Novelty of our case is that we successfully rechallenged the patient with the same chemotherapy regimen cisplatin and etoposide under the cover of antiplatelet. The decision for rechallenging was taken based on his young age, no comorbid condition, and limited stage disease and to cure the disease. The decision to rechallenge should be made after assessing the risk and benefit depending on a case‐by‐case basis.

Patients with risk factors such as old age, previous episodes of TE, smoking and medical condition predisposing to thromboembolism, might be more prone to chemotherapy related vascular toxicity and they should be monitored carefully, and modifiable risk factors should be addressed before and during the treatment. Besides, a recent study notified that the use of low molecular weight heparin (LWMH) resulted in a significant reduction in incidence of thrombotic events.^15^ Nonetheless, further studies need to be done to design proper guidelines for thromboprophylaxis therapy in cancer patients.

## 4 CONCLUSION

In conclusion, we report a case of cerebrovascular ischemic event followed by cisplatin‐based chemotherapy for limited‐stage SCLC. Workup for potential sources of CVA like cardiovascular causes was negative and the only risk factor patient had been smoking history and the cisplatin‐based chemotherapy. Therefore, emergency physicians, neurologists, cardiologists, and as well as oncologists need to be aware of this potential side effect of cisplatin for any patient who is presenting with acute chest pain or neurological deficit. In addition, oncologists should be vigilant in initiating chemotherapy and work in collaboration with primary care physicians to address modifiable risk factors such as diabetes, hypertension, and dyslipidemia to possibly reduce the incidence of cisplatin‐induced vascular toxicity.

## AUTHOR CONTRIBUTIONS

FR and NEO contributed equally to this work; they conceived and designed the idea, literature review, data collection, and wrote the initial manuscript draft. HA and OA contributed to the initial manuscript draft. UH helped in reviewing the initial draft and finalizing it. FR and AS helped in the radiology figures and helped in manuscript writing. NEO contributed to the manuscript writing and organize the overall case report. All authors critically reviewed the article, gave final approval of the version to be published and agreed to be accountable for all aspects of the work.

## FUNDING INFORMATION

Open Access funding provided by the Qatar National Library.

## CONFLICT OF INTEREST

The authors declare that there was conflict no of interest regarding the publication of this case report.

## ETHICAL APPROVAL

The case report was approved by the Medical Research Centre at Hamad Medical Corporation and the Hamad Institutional Review Board (IRB) under number 0422‐260.

## CONSENT

This case report does not contain any personal identifier of the patient (such as name and photograph). It only includes radiological and pathological imaging, which does not contain any identification. A written patient informed consent of patient information, images and publication was signed by the patient.

## Data Availability

The datasets used and/or analyzed during the current study are available from the corresponding author on reasonable request.
